# Advances and limitations of semi-elastic pneumatic cuffs in blood flow restriction training: a narrative review

**DOI:** 10.3389/fphys.2025.1632367

**Published:** 2025-09-03

**Authors:** Sten Stray-Gundersen, James Stampley

**Affiliations:** Department of Exercise Science, Arnold School of Public Health, University of South Carolina, Columbia, SC, United States

**Keywords:** blood flow restriction training, multi-chambered cuffs, wide-rigid cuffs, arterial occlusion pressure, resistance training, aerobic training, cardiovascular, perceptual responses

## Abstract

Semi-elastic pneumatic (SEP) blood flow restriction (BFR) cuffs, such as the original KAATSU and the B Strong/B3 cuffs, have gained popularity as practical, user-friendly tools for low-load BFR exercise. However, their efficacy and optimal use remain somewhat debated, especially compared to rigid tourniquet-style cuffs that prescribe individualized arterial occlusion pressures (AOPs). This narrative review synthesizes the literature on SEP BFR devices with a focus on B Strong/B3 to clarify their acute and chronic effects and address unique methodological considerations. We first define SEP BFR cuffs and distinguish them from rigid AOP-calibrated cuffs, then summarize the acute and chronic physiological and perceptual responses and adaptations to resistance and aerobic exercise. Acutely, SEP BFR cuffs elicit pronounced local metabolic stress and fatigue. Longitudinally, SEP BFR training leads to significant improvements in muscle size, strength, and endurance. SEP BFR cuffs may offer practical advantages in safety and accessibility to a wide range of users by using a design that inherently limits the risk of arterial occlusion. We also discuss practical considerations for SEP BFR, propose alternative internal load monitoring such as near-infrared spectroscopy, and emphasize that the degree of fatigue and effort are primary hallmarks of an effective BFR training session. Finally, we propose future directions for research along with considerations on how to optimally apply and study SEP BFR. While SEP BFR cuffs are designed to not fully occlude arterial flow—thereby limiting their capacity for AOP standardization—they offer a pneumatically-controlled approach capable of delivering a safe and effective BFR training stimulus. Given their growing use in the field, researchers should not dismiss SEP BFR devices; instead, they should be systemically investigated and undergo direct comparisons with rigid AOP-based devices. Such research will help refine guidelines and broaden our understanding of how both SEP and AOP-calibrated BFR can be optimally applied.

## 1 Introduction

Blood flow restriction (BFR) training involves exercising with cuffs placed on the proximal portion of the limbs to restrict blood flow typically during low-load or low-intensity exercise (Scott et al., 2015). The primary goal of BFR is to partially restrict arterial inflow and intermittently occlude venous outflow from the limb in order to amplify metabolic stress and accelerate fatigue during exercise. Passive forms of BFR, such as ischemic preconditioning (Kharbanda et al., 2001) and tissue flossing (Tedeschi and Giorgi, 2024) have also gained attention (Patterson and Brandner, 2018; Patterson et al., 2019), but this review will primarily focus on exercise with BFR.

While the modern BFR research landscape is dominated by rigid nylon pneumatic cuffs calibrated to an individual’s arterial occlusion pressure (AOP) (Loenneke et al., 2011; Patterson et al., 2019), this represents somewhat of a departure from its original form. BFR training was first developed in Japan by Dr. Yoshiaki Sato as the KAATSU method, which utilizes a semi-elastic pneumatic (SEP) cuff and a stepwise progression of pressure (“KAATSU cycle”) to prime the vasculature and avoid full arterial occlusion during training (Patterson et al., 2019; Sato, 2005). Importantly, while KAATSU systems can achieve arterial occlusion, the AOP approach is generally not recommended in their protocols. Instead, KAATSU recommends protocols such as assessing capillary refill time by pressing on the palm or foot to determine the pressure needed to adequately restrict arterial flow and prevent complete occlusion. In contrast, modern SEP BFR devices such as B Strong and B3 have been developed to emulate the SEP approach of KAATSU but were designed to avoid arterial occlusion, utilize preset inflation guidelines for training, and offer simplified application (Early et al., 2020; Stray-Gundersen et al., 2020; Citherlet et al., 2022). For clarity, B Strong and B3 utilize the same cuff design but represent two separate companies distributing BFR products. While B Strong/B3 and KAATSU share the foundational SEP concept, they differ in design, procedures, and real-world deployment.

To further highlight differences in BFR cuff design and application, it is important to provide historical context. As interest in BFR training increased in the early 2000s, Western researchers sought to study its physiological effects by using equipment available in their laboratories, primarily inelastic nylon blood pressure cuffs primarily used for vascular assessments. These devices allow for complete arterial occlusion and limb pressure standardization across individuals and laboratories. This shift, while scientifically pragmatic, marked a methodological deviation from the original KAATSU approach. Then, as the use of BFR became more widespread, other SEP BFR devices (e.g., B Strong/B3) were developed to mimic the original SEP intent when using KAATSU while attempting to simplify the application process.

**FIGURE 1:**
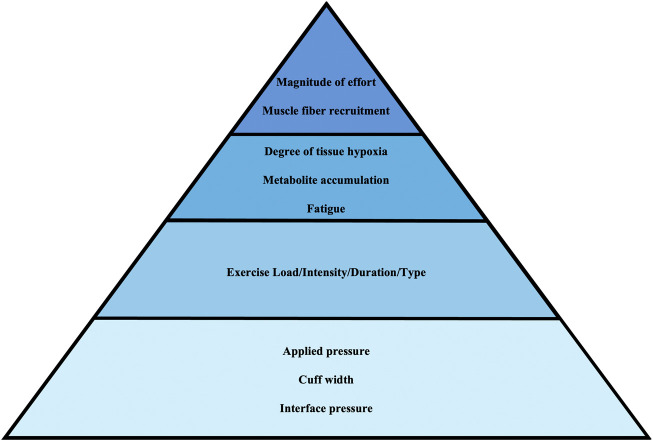
Proposed hierarchy of important BFR factors and stimuli for adaptation (top = more important; bottom = less important).

Despite these factors, SEP BFR cuffs are underrepresented in recent BFR literature presumably due to their inability to support AOP-based calibration. Existing reviews tend to emphasize rigid cuff designs or focus heavily on AOP as a standardization tool, potentially dismissing the broader spectrum of effective BFR methods. Indeed, previous articles (Patterson and Brandner, 2018; Rolnick et al., 2023; Rossow et al., 2012) have highlighted that failure to individualize pressure settings by not using AOP-based calibration can affect the nature of the perceptual, neuromuscular, hemodynamic, and metabolic responses as well as the physiological adaptations that follow while a recent editorial emphasized that BFR methods and apparatuses still matter and should be carefully matched to the population and goals (Hughes et al., 2025). In addition, the notion of interface pressure, defined as the pressure exerted by the cuff on the surface of the limb has been raised as a potential confounding factor in BFR pressure prescription (Rolnick et al., 2023; Rolnick et al., 2025a). While these are valid critiques and recommendations, it is important not to discourage investigations using SEP BFR devices. Unlike rigid AOP-based cuffs that aim to completely stop arterial inflow to determine AOP, SEP BFR cuffs aim to avoid arterial occlusion, limit peak pressures during muscle contraction, and minimize perceptual and cardiovascular strain (Loenneke et al., 2011; Stray-Gundersen et al., 2020). In addition, SEP BFR devices are generally accessible for a variety of applications and allow for multi-limb exercise, making them ideal for use in gyms, clinics, home environments, and populations that may be most likely to benefit from BFR training. While AOP-based approaches offer enhanced precision and standardization, their practical implementation is limited by variability in cuff size/width, numerical pressures employed, and design. One example has been highlighted recently—practitioners employ pressures from published studies using different sized cuffs which may alter the physiological effects despite matching the numerical pressure value (Patterson and Brandner, 2018). In addition, some AOP-based devices have demonstrated substantial variability in maintaining target pressures during training (Swain et al., 2025). Considering these variable factors, all features of a given BFR cuff and protocol should be highlighted when reporting findings. At the same time, there may be an overemphasis on the cuff pressures applied at the expense of the primary target of BFR training—accelerating fatigue and enhancing motor unit recruitment during an exercise movement (Figure 1). For example, two separate laboratories applying 50% AOP could observe different outcomes if one uses lower effort and the other pushes to volitional failure; conversely, using different pressures (e.g., 40% *versus* 60% AOP) might yield similar adaptations if both protocols induce comparable levels of fatigue and effort. This perspective is supported by a recent meta-analysis comparing various BFR repetition-set schemes to traditional resistance training, which concluded that when effort is matched, the resulting adaptations are largely similar regardless of the specific repetition-set scheme used (De Queiros et al., 2024). Therefore, emphasis on precise cuff pressure without context may divert attention from the more relevant drivers of adaptation: the degree of fatigue, muscle recruitment, and effort. Rather than dismissing SEP BFR for its inability to fully occlude, it should be systemically investigated and compared directly to AOP-based systems to elucidate differences in applications, responses, and outcomes.

In the sections that follow, we further clarify how SEP BFR devices differ from rigid AOP-based cuffs. While our primary focus is on comparisons between SEP BFR cuffs and rigid AOP-based devices, we must also acknowledge the rapidly growing use of effective practical BFR methods such as knee wraps and non-pneumatic elastic straps. In addition, while KAATSU can be categorized as a SEP BFR device, this review will primarily focus on studies utilizing SEP BFR cuffs like B Strong or B3, as there is a dearth of studies investigating this specific type of SEP BFR cuff. To the authors’ knowledge, no focused narrative review has evaluated the acute and chronic physiological effects, methodological considerations, and practical applications of SEP BFR devices. Accordingly, this review aims to: 1) highlight acute and chronic physiological responses specific to SEP BFR, 2) identify methodological considerations unique to SEP BFR devices, and 3) encourage further comparative research in this relatively underexplored area. Our aim is to provide a balanced, evidence-based perspective that encourages thoughtful integration of SEP BFR into practice and research.

## 2 Defining SEP vs. AOP-Based cuffs

**FIGURE 2:**
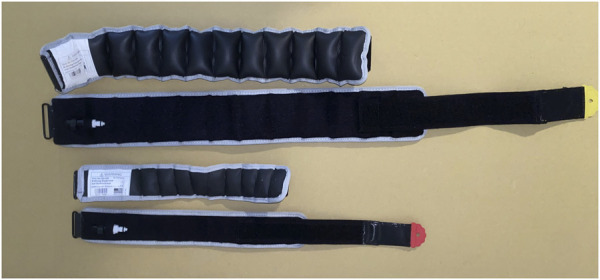
Examples of leg (top) and arm (bottom) SEP BFR (B Strong) cuffs inflated (top) and deflated (bottom) to illustrate the barrel-/accordion-like design.

SEP BFR cuffs incorporate semi-elastic and pneumatic components that allow them to stretch and expand with muscle contraction while enabling a repeatable application across individuals and sessions. For example, the KAATSU cuff consists of a 4–6 cm wide semi-elastic cuff with an inflatable bladder, while the B Strong/B3 cuffs incorporate a 5–7.5 cm barrel-/accordion-like cuff design often described as “multi-chambered” (MC). Studies show that when inflated to recommended pressures (e.g., 250–350 mmHg for the legs), these designs generate substantial external compression and can restrict arterial blood flow but do not aim to occlude the arteries (Citherlet et al., 2022). This is made possible by their barrel-/accordion-like structure (Figure 2), which allows an otherwise rigid cuff to become semi-elastic when inflated. This semi-elasticity may enhance safety and comfort compared to inelastic cuffs, particularly when such cuffs are not calibrated to an individual’s AOP (Chen L. et al., 2025; Citherlet et al., 2022; Rolnick et al., 2023). In contrast, rigid cuffs (e.g., Hokanson) lack material compliance to accommodate muscle expansion during contraction and are designed to occlude arteries.

The semi-elastic nature of SEP BFR cuffs means the applied pressure is partially self-regulating as the cuffs can accommodate dynamic muscle expansion during exercise, which may help to attenuate pressure spikes, retrograde shear stress, cardiovascular strain, and overall discomfort (Chen L.-S. et al., 2025; Citherlet et al., 2022; Stray-Gundersen et al., 2020). Additionally, it is important to distinguish MC cuffs, which would have separate inflation compartments that can be cycled sequentially from the so-called “MC” B Strong/B3 cuff. The B Strong/B3 cuff’s internal air channels are interconnected and inflate simultaneously as a single unit, functioning effectively as one bladder that wraps around the limb. Thus, while it has the appearance of multiple air cells, it does not provide graded sequential compression; rather, the design allows some degree of elasticity due to its barrel-/accordion-like design when inflated (Figure 2). This design serves to displace the pressure during peak concentric muscle contraction throughout the cuff rather than directly into the limb and underlying vasculature and nerves. Importantly, the elasticity of SEP BFR cuffs is also affected by the inflation pressure. Above a threshold (∼250 mmHg), the cuff becomes increasingly stiff, offering slightly less compliance and a greater magnitude of blood flow restriction. This property allows practitioners to adjust and progress SEP BFR cuff pressures based on the user’s muscle size, age, or pressure tolerance, offering some degree of pressure individualization in the absence of AOP calibration. While two studies were able to occlude arterial flow during seated rest using B3 cuffs (Wilburn et al., 2021; Machek et al., 2023), others (Citherlet et al., 2022; Chen L. et al., 2025; Chen L.-S. et al., 2025; Montoye et al., 2023) have been unable determine AOP when using B Strong cuffs. Moreover, while increasing SEP BFR cuff pressure from 200 to 400 mmHg reduced resting arterial blood flow (albeit inconsistently), beyond a certain threshold, further increases in pressure did not produce additional reductions in flow (Citherlet et al., 2022). By contrast, a rigid cuff calibrated to a percentage of AOP (e.g., 40% or 60%) can produce graded flow reductions, though these reductions are not linear (Citherlet et al., 2022; Mouser et al., 2017).

Importantly, while the AOP approach is conceptually appealing, its practical implementation has limitations that should be addressed. Blood pressure and vascular hemodynamics change on a beat-to-beat basis during both rest and exercise (Macdougall et al., 1985), and AOP, typically measured at rest in a seated or supine position, does not linearly correspond to reductions in arterial blood flow (Citherlet et al., 2022; Mouser et al., 2017). Moreover, a primary purpose of using a percentage of resting AOP is to reduce the risk of arterial occlusion during exercise (Clarkson et al., 2020; Patterson et al., 2019). However, the pressure required to occlude arterial blood flow during dynamic activity is markedly higher than at rest, and a set percentage of AOP does not uniformly reduce flow across individuals, and likely not across sessions in the same individual due to variations in hemodynamics based on time of day, hydration status, and other transient physiological factors (Schutte et al., 2022). Thus, while AOP-based calibration introduces a useful framework for relative standardization at rest, its application may not fully capture the dynamic and individualized nature of hemodynamic responses during exercise. Moreover, a range of AOP-based pressures (40%–80%) has been deemed safe and effective (Patterson et al., 2019), but these different pressures elicit distinct fatigue rates and magnitudes of physiological stress (Fatela et al., 2016). The “ideal” BFR pressure likely depends on several variables, including population characteristics, exercise modality, limb size, and cuff width (Clarkson et al., 2020). Given that high pressures with wide (10–18 cm) cuffs are not required to achieve meaningful adaptations (Loenneke et al., 2012) and may elevate perceptual responses or attenuate muscle growth directly beneath the cuff (Ellefsen et al., 2015; Kacin and Strazar, 2011), practitioners may prefer to apply modest pressures with medium-width cuffs (5–10 cm) to minimize discomfort from the cuff itself. Additionally, while cuff width remains an important consideration, in this review we only differentiate wide-rigid (WR) from rigid cuffs when a study explicitly identifies them as such.

## 3 Acute responses and chronic adaptations to SEP BFR exercise

The ultimate question for any training modality is whether it produces meaningful acute responses and chronic adaptations. Acute physiological responses to SEP BFR exercise include changes in muscle activation, hemodynamics, metabolic accumulation, and perceptual measures during and immediately after a training session. Indeed, SEP BFR cuffs markedly enhance local muscle fatigue and metabolic stress compared to the same exercise without BFR while producing acute cardiovascular responses similar to non-BFR exercise (Stray-Gundersen et al., 2020; Wooten et al., 2020). These acute effects underlie the significant long-term improvements observed in muscle strength, hypertrophy, endurance, and vascular function. In the sections that follow, we review both acute and longitudinal findings from resistance and aerobic training studies, highlighting key outcomes and protocol considerations specific to SEP BFR devices.

### 3.1 Resistance exercise

Applying SEP BFR cuffs during low-load resistance exercise precipitates an earlier onset of fatigue and augments motor-unit recruitment compared to load- and volume-matched controls (Bordessa et al., 2021; Dancy et al., 2023; Rolnick et al., 2025b; Rolnick et al., 2024). In a crossover trial, Dancy et al. compared rigid AOP-based devices (Delfi PTS, SmartCuffs) with a SEP BFR (B Strong) cuff during two sets of bicep curls at 20% one-repetition maximum (1RM) performed to failure in 21 healthy adults; all BFR conditions shortened time to failure *versus* control, but the SEP BFR cuffs permitted a similar repetition count while provoking lower perceived exertion until the final set (Dancy et al., 2023). Likewise, Bordessa et al. reported equivalent quadricep electromyography (EMG) amplitudes during knee extensions at 30% 1RM with a SEP BFR (B Strong) cuff set between 250 and 310 mmHg and a rigid cuff set at 30% AOP in 34 healthy adults, yet the rigid cuff elicited significantly greater ratings of perceived exertion (RPE) and pain scales likely from the mechanical compression induced by the rigid cuff (Bordessa et al., 2021).

In contrast, recent work by Rolnick and colleagues showed that MC/SEP BFR (B Strong) cuffs did not accelerate wall-squat repetitions to failure as rapidly as rigid single-chambered (SC) AOP-based (Delfi PTS) cuffs (SC: 57 vs. MC/SEP: 76), but did accelerate repetitions to failure more rapidly than no BFR (106) in 27 resistance-trained adults (Rolnick et al., 2024). Notably, users experienced similar levels of discomfort in both BFR groups compared to no BFR. However, the use of the Delfi PTS cuffs improved acute measures of central arterial stiffness whereas the MC/SEP and non BFR conditions increased central arterial stiffness. In a separate randomized crossover trial using upper-body exercise, Rolnick et al. compared the perceptual and cardiovascular responses of a rigid SC AOP-based (Delfi PTS) cuff and an MC/SEP BFR (B Strong) cuff during four sets of bilateral bicep curls to failure in 26 resistance-trained adults. The SEP BFR cuffs did not accelerate repetitions to failure as rapidly as the rigid SC AOP-based cuffs until the final set, but produced lower perceived discomfort, underscoring the influence of cuff design on perceptual responses (Rolnick et al., 2025a). Importantly, this may suggest that higher pressures accelerate fatigue more rapidly, which may be an important consideration for BFR training. However, taken together, these findings suggest that SEP BFR cuffs can deliver a robust training stimulus by reducing repetitions to failure albeit less rapidly than some rigid AOP-based systems.

SEP BFR has also been shown to markedly amplify metabolite accumulation during resistance exercise tasks. An investigation conducted in 16 collegiate swimmers revealed that back squats performed at 30% 1RM with SEP BFR (B Strong set at 200 mmHg) elevated post-exercise blood lactate to levels similar to traditional high-load (70% 1RM) sessions (Wang et al., 2023). Chen, Brosselin, et al. also showed similar increases in blood lactate between WR (GF Health Products) and SEP BFR (B Strong) cuffs following a series of upper body exercises in 26 young healthy adults (Chen L. et al., 2025). In addition, a single-subject MRI case study showed pronounced intramuscular fluid shifts and sarcomere swelling following unilateral leg-press at 30% 1RM performed with SEP BFR (B3) cuffs (Wilburn et al., 2021). While Wilburn et al. reported that BFR-induced fluid pooling acutely altered skeletal muscle ultrastructure compared to traditional heavy training, these studies collectively demonstrate that SEP BFR cuffs enable low-intensity resistance exercise to produce a pronounced metabolic stimulus.

Additionally, acute cardiovascular strain appears moderate with SEP BFR cuffs during resistance exercise. Wooten et al. found that combining SEP BFR (B Strong) with an isometric exercise (yoga) elicited only mild pressor responses that were no different than the non-BFR condition in 20 young healthy adults, supporting the cardiovascular safety of SEP BFR cuffs during isometric exercise, which can elicit substantial transient increases in blood pressure (Wooten et al., 2020). Chen, Brosselin, et al. compared blood pressure and physiological responses before and after three sets of single-arm bicep curls (40% 1RM), triceps extensions (40% 1RM), and handgrip exercise (60% MVC) with a WR (GF Health Products) cuff (set at 80 mmHg) and narrow-elastic (NE)/SEP (B Strong) cuff (set at 220 mmHg) in 26 healthy adults. When compared to NE/SEP BFR cuffs, WR cuffs elicited higher mean arterial pressures across all exercises. In addition, perceived exertion and pain responses were greater in the WR cuff condition while maintaining similar heart rate responses and blood lactate concentrations to the NE/SEP BFR cuff condition (Chen L.-S. et al., 2025). However, regarding differences in exercise performance, RPE, and safety, conflicting results have been reported across multiple studies. In a randomized crossover trial involving 21 healthy adults, Dancy et al. found no significant differences in exercise performance, RPE, or safety across three commonly-used BFR cuffs during resistance exercise, including both SEP (B Strong) and AOP-based (Delfi PTS, SmartCuffs) BFR cuffs (Dancy et al., 2023). In contrast, two recent randomized crossover trials by Rolnick and colleagues (Rolnick et al., 2024; Rolnick et al., 2025b), demonstrates that SEP BFR (B Strong) cuffs consistently elicit lower perceived discomfort than rigid AOP-calibrated (Delfi PTS) cuffs during resistance training. These findings, observed across different laboratory settings and participant samples, underscore the critical impact of cuff design on both the physiological and perceptual responses to BFR exercise. Collectively, however, these findings support the perceptual tolerability of SEP BFR cuffs during resistance exercise.

Acute resistance exercise with SEP BFR cuffs therefore produces 1) enhanced motor-unit recruitment during low-load training, 2) robust metabolic perturbation evidenced by high blood lactate and cellular swelling, 3) pressor responses similar to non-BFR exercise, and 4) lower pain and discomfort relative to rigid cuffs, particularly if they are not calibrated to an individual’s AOP. These characteristics make SEP BFR a practical strategy for eliciting high-intensity local stimuli with low loads while minimizing cardiovascular and perceptual strain.

Several trials have also examined weeks of low-load resistance training with SEP BFR cuffs (Chen L. et al., 2025; Cintineo et al., 2024; Early et al., 2020; Wooten et al., 2022). Overall, studies report robust increases in muscular strength and size, comparable in magnitude to traditional high-load training. Perhaps most notably, Early et al. conducted an 8-week randomized trial in 31 young adults comparing three groups: high-load resistance training (60% 1RM) *versus* low-load resistance training with SEP BFR (B Strong) cuffs (30% 1RM set at 250 mmHg for arms and 350 mmHg for legs) *versus* a no-exercise control (Early et al., 2020). At the end of 8 weeks, the high-load and SEP BFR groups significantly increased muscle strength and endurance on a variety of exercises with no significant difference between groups (Early et al., 2020). Importantly, Early et al. also measured vascular function via brachial artery FMD, and found that both the high-load traditional and low-load SEP BFR training led to significant increases in FMD relative to the no-exercise control (Early et al., 2020). Additionally, the investigators monitored muscle soreness throughout the training program: both groups reported similar soreness during the program except for the final week, in which the SEP BFR group reported less muscle soreness 24 h post training than the high-load group (Early et al., 2020). The authors concluded that BFR is an effective alternative to high-load training to elicit gains in muscle strength, endurance, and vascular function (Early et al., 2020).

A recent finding by Chen, McLaurin et al. also points to the safety and efficacy of SEP BFR (Chen L.-S. et al., 2025). Twenty-six participants performed 2 weeks of work-load matched arm resistance training with one arm using a NE/SEP (B Strong) cuff and the other arm using a WR (GF Health Products) cuff, allowing a within-subject comparison (Chen L. et al., 2025). Despite both arms increasing muscle strength, each arm displayed slightly different vascular adaptations: the arm trained with the SEP BFR cuff showed a significant improvement in brachial FMD (5.6% to 7.7%) whereas the arm trained with the WR cuff exhibited a non-significant decrease in FMD (6.0% to 4.9%) (Chen L.-S. et al., 2025). The researchers linked these adaptations to the differential hemodynamic patterns during exercise: WR cuffs exhibited higher retrograde shear rate, which may have impaired endothelial function (Chen L. et al., 2025). Importantly, the WR condition did not employ AOP-based calibration or autoregulation, which may have contributed to the increases in retrograde shear rate and to the lack of vascular improvement when accounting for previous findings (Rolnick et al., 2024). Although longer and larger trials are still needed, Chen, McLaurin et al.’s short-term observations are consistent with previous studies utilizing other SEP BFR (KAATSU) cuffs, which have repeatedly been shown to promote improvements in vascular function (Christiansen et al., 2019; Larkin et al., 2012; Shimizu et al., 2016).

Another noteworthy study is from Cintineo et al., who implemented a 6-week training program in 54 U.S. Army ROTC cadets. Participants were randomized to a 4-day per week traditional resistance training program, a minimal equipment training program using body-weight, sandbags, and resistance bands, or the same minimal equipment program with SEP BFR (B Strong) cuffs (Cintineo et al., 2024). The results showed that all groups improved performance across a battery of fitness assessments (vertical jump, bench press, VO_2_max), with the traditional high-load group showing the greatest gains in maximal strength (Cintineo et al., 2024). Importantly, minimal equipment training with and without BFR exhibited higher relative effort levels (higher heart rates, blood lactate, and RPE) than the traditional training group (Cintineo et al., 2024), highlighting a critical point in BFR application: when effort is high and training approaches failure, performance outcomes can be similar with or without BFR.

Additional longitudinal research further supports the efficacy of SEP BFR for enhancing performance adaptations. Zhou et al. conducted a randomized controlled trial in 26 University athletes, in which complex resistance training incorporating plyometrics with SEP BFR (B Strong) over 4 weeks resulted in significantly greater improvements in power output and bar velocity during half-squat jumps compared to traditional training (Zhou et al., 2024). These findings suggest that SEP BFR can augment neuromuscular adaptations when integrated into performance-focused resistance programs. In addition, 4 weeks of core-focused SEP BFR (B Strong set at 180 mmHg for arm and legs) training produced session RPE and heart rate responses comparable to high-load training in 26 young male athletes with chronic low-back pain (Liu et al., 2025). Similarly, Wang et al. showed improvements in leg strength on par with high-load resistance training in 16 young swimmers undergoing a 4-week resistance training program with (n = 8) and without (n = 8) SEP BFR (B Strong set at 200 mmHg) (Wang et al., 2023).

In addition, in 16 male sprinters, single bouts of SEP BFR (BStrong set between 200 and 350 mmHg), whole-body vibration (WBV), or their combination (SEP BFR + WBV) each improved 20 m sprint performance, with SEP BFR + WBV and WBV alone enhancing 10 m performance (Zhang et al., 2023). SEP BFR + WBV also increased EMG amplitude in the vastus lateralis and soleus muscles, while SEP BFR alone elicited greater tibialis anterior activation and higher post-exercise blood lactate than the combined protocol, suggesting distinct neuromuscular and metabolic effects depending on the application mode. Moreover, Wang et al. employed an 8-week half-squat training program in 18 male collegiate volleyball players (Wang et al., 2022). The researchers randomly assigned participants to one of three groups: high-load (70% 1RM, n = 6) with SEP BFR (B Strong set at 180 mmHg), low-load (30% 1RM, n = 6) with SEP BFR (B Strong set at 180 mmHg), and high-load (70% 1RM, n = 6) without BFR. Notably, the group that combined high-load training with BFR demonstrated the greatest improvements in both strength and vertical jump compared to the low-load BFR group, and while their gains exceeded those of the high-load group, the differences were not statistically significant (Wang et al., 2022). This suggests a possible synergistic effect of using high loads with SEP to elicit robust adaptations in athletic populations, though cautious interpretation is warranted given that BFR combined with low loads (20%–40% 1RM) is generally recommended (Loenneke et al., 2011; Patterson et al., 2019).

Finally, in a randomized, placebo-controlled study, Machek et al. used SEP BFR (B3) cuffs to determine AOP and assigned 18 recreationally trained males to supplement with either 6 g/day of betaine anhydrous or a cellulose placebo for 14 days (Machek et al., 2022). Participants then performed four standardized sets of one-leg leg press followed by two additional sets to failure on both legs, using low-load BFR (20% 1RM at 80% AOP) on one leg and high-load (70% 1RM) on the other. The high-load group elicited a significantly greater change in blood lactate compared to low-load BFR and betaine supplementation augmented post-exercise insulin-like growth factor 1 concentrations relative to placebo, while the high-load group exhibited a greater change in serum homocysteine than the low-load BFR group (Machek et al., 2022). Taken together, these findings highlight that SEP BFR training can enhance strength, power, and neuromuscular adaptations across diverse training modalities and populations, with potential additive effects when paired with high-load resistance exercise, though further work is needed to refine its optimal application parameters.

SEP BFR training has also shown efficacy in clinical settings. Wooten et al. implemented a 4-week multimodal prehabilitation program for 24 abdominal cancer patients awaiting surgery that included a home-based SEP BFR (B Strong) training program plus nutritional supplementation consisting of whey protein, l-citrulline, and creatine (Wooten et al., 2021). The multimodal prehabilitation program significantly increased total lean body mass (45.2 ± 12.3 kg to 46.0 ± 12.2 kg) and decreased total fat mass (36.0 ± 10.7 kg to 35.3 ± 10.7 kg) while remaining weight stable (75.3 ± 19.2 kg to 75.5 ± 18.6 kg). In addition, patients significantly improved their 6-min walk test (+48 ± 53 m), time to complete the 5-repetition chair stand test (pre: 14.6 ± 11.4 s vs. post: 9.8 ± 3.8 s), timed up and go test (−0.90 ± 0.72 s), but did not improve handgrip strength (pre: 28.6 ± 11.7 kg vs. post: 28.8 ± 13.5 kg). In a follow-up cohort study, the patients who underwent the SEP BFR prehabilitation + nutritional intervention exhibited shorter postoperative hospital stays and fewer surgical complications when compared to retrospective data on 71 abdominal cancer patients who underwent usual preoperative care (Wooten et al., 2022). While this was a multifaceted intervention that lacked a work-matched control group, the inclusion of BFR exercise was highlighted as a key component enabling frail patients to exercise at sufficient intensity to confer meaningful functional improvements. Such findings highlight that SEP BFR training can be applied in real-world clinical scenarios to improve patient outcomes.

Overall, the limited data on long-term resistance training with SEP BFR suggests significant hypertrophy and strength gains in healthy and clinical populations. However, more long-term training studies are necessary to corroborate these findings.

### 3.2 Aerobic exercise

Aerobic endurance exercise (e.g., walking, running, cycling) performed with SEP BFR cuffs can heighten the internal load of otherwise light work while imposing a modest cardiovascular strain. Despite a strong safety profile established in Japan when using the original KAATSU device (Nakajima et al., 2006), early concerns surrounding BFR training, particularly in at-risk populations, focused largely on the potential for exaggerated cardiovascular responses (Eiken and Bjurstedt, 1987; Spranger et al., 2015). The seminal work from Alam and Smirk in the 1930s established that reductions in blood flow to exercising muscle engage the exercise pressor reflex, leading to excessive increases in blood pressure (Alam and Smirk, 1937). Put in the context of BFR and the early work by Eiken and Bjurstedt, the fear was that restriction of arterial blood flow during even low-intensity exercise may impose excessive cardiovascular strain and negatively impact vascular function. Therefore, Renzi et al. investigated the acute responses of 17 young healthy participants to low-intensity walking with WR BFR (Hokanson) thigh cuffs set to 160 mmHg (AOP not measured) and observed elevated heart rate, blood pressure, and rate-pressure product compared to unrestricted walking (Renzi et al., 2010). Importantly, they also observed a marked decline in popliteal artery FMD following BFR walking, indicating acute endothelial dysfunction presumably via ischemia-reperfusion insult. These findings fueled concerns surrounding the safety of BFR in individuals with compromised cardiovascular health.

To address these concerns and differentiate between cuff types, Stray-Gundersen et al. conducted a follow-up study evaluating the acute cardiovascular responses during low-intensity walking with either WR (Hokanson) or NE/SEP BFR (B Strong) cuffs in 15 young healthy adults as NE/SEP BFR cuffs were hypothesized to induce less pronounced pressor responses (Stray-Gundersen et al., 2020). Stray-Gundersen et al. reported that the NE/SEP BFR cuff inflated to 300 mmHg produced increases in heart rate and systolic blood pressure that were similar to unrestricted walking, whereas the WR cuffs set to 160 mmHg (AOP not measured) provoked greater pressor and RPE responses. Notably, neither cuff condition raised measures of central arterial stiffness or impaired vascular function. While these results should be interpreted in the context of lower blood lactate levels observed in the NE/SEP condition, which may have attenuated the pressor response, Sullivan et al. has demonstrated that low-intensity treadmill running (∼40% VO_2_max) with SEP BFR (B Strong) cuffs elevate post-exercise blood lactate and RPE to levels comparable to high-intensity exercise (∼80% VO_2_max) in 15 female distance runners (Sullivan et al., 2025). Notably, heart rate increased moderately compared to the same exercise without BFR but was lower than high-intensity exercise. While the methods for determining pressure may have been suboptimal (assessing AOP with a rigid Hokanson cuff, then applying those pressures to a SEP BFR (B Strong) cuff (Rolnick et al., 2025a), the findings highlight that SEP BFR can elicit a robust physiological stimulus. Collectively, these investigations underscore that AOP-based calibration is essential when using rigid BFR cuffs to ensure both safety and efficacy.

Additionally, Landers et al. conducted a non-crossover trial in 18 healthy adults (n = 10: BFR, n = 8: control) and showed that 15 min of low-intensity arm and leg cycling with SEP BFR (B Strong) cuffs tended to increase tissue-plasminogen-activator activity immediately post-exercise compared to the same exercise without BFR (BFR: 292.57 ± 448.37 pg/mL vs. control: 30.54 ± 108.24 pg/mL) (Landers et al., 2025). Although the sample size was small, the observed effect size (partial eta = 0.14) and trend toward enhanced fibrinolytic potential suggest that SEP BFR does not impair, and may even stimulate, the fibrinolytic system during aerobic work compared to the same exercise under non-BFR conditions. In addition, Callanan et al. implemented a vertically oriented, full-body exercise modality using the VersaClimber to determine the systemic effects of BFR. Fifteen active adults completed 9 minutes on the VersaClimber either with or without SEP BFR (B Strong) cuffs applied to all four limbs. Despite performing identical workloads, the SEP BFR group exhibited higher blood lactate concentrations and RPE, demonstrating a greater internal training load stimulus compared to control. Importantly, both sessions lead to significant increases in CD34^+^, a marker for hematopoietic progenitor cells, with no significant differences between groups (SEP BFR: 38.7% vs. control: 33.3% increase), suggesting a lack of a cellular systemic response from BFR. Nonetheless, this study highlights the potential of multi-limb SEP BFR training to enhance metabolic stress in whole-body training contexts. Taken together, these studies suggest that SEP BFR can provide a meaningful training stimulus safely during aerobic exercise.

Even fewer longitudinal studies exist for aerobic training with SEP BFR, but initial results are encouraging. Low-intensity aerobic exercise with SEP BFR have been shown to improve VO_2_max and endurance performance, approaching adaptations seen with higher-intensity training without BFR. Mechanistically, the enhanced metabolite accumulation and hypoxic stimulus from aerobic BFR training can trigger peripheral adaptations such as increased capillarization and mitochondrial enzyme expression via upregulation of vascular endothelial growth factor (VEGF) and peroxisome proliferator-activated receptor gamma coactivator 1-alpha (PGC-1α) expression following SEP BFR (KAATSU) aerobic protocols (Christiansen et al., 2019; Larkin et al., 2012). Additionally, SEP BFR aerobic training can yield improvements in functional capacity and cardiovascular health across diverse populations. As mentioned previously, Wooten et al. reported that in abdominal cancer patients, a home-based SEP BFR exercise program not only enhanced surgical outcomes (Wooten et al., 2022) and improved performance on functional fitness tests such as the timed-up-and-go and sit-to-stand, but also significantly increased distance covered in the 6-min walk test (Wooten et al., 2022), a robust indicator of aerobic capacity in clinical populations. These findings are consistent with a recent meta-analysis by Gao et al., which synthesized results from 16 BFR aerobic training studies and demonstrated that aerobic BFR training significantly improves both VO_2_max and muscle strength compared to traditional aerobic training (Gao et al., 2025).

In summary, current limited evidence indicates that SEP BFR can effectively enhance both aerobic and resistance training outcomes, with adaptations spanning strength, hypertrophy, endurance, and vascular function across a variety of populations. Its practical safety profile, accessibility, and versatility further support its application in both clinical and performance contexts (Callanan et al., 2022; Wooten et al., 2022; Zhou et al., 2024). Ongoing research should prioritize head-to-head comparisons and mechanistic studies to fully elucidate any differences in adaptations observed between SEP BFR and AOP-calibrated devices. A consolidated table summarizing study design, participant characteristics, exercise type, pressure settings, and key findings in studies using SEP BFR is included in Table 1.

**TABLE 1:** Human studies employing semi-elastic pneumatic (SEP) blood-flow-restriction (BFR) devices.

Citation (author et al. year)	Design (N)	Population	Exercise type	Devices/Pressure	Key findings
Chen et al. (2025b)	Acute crossover (n = 26)	Young adults	Arm exercises	WR cuff (GF Health Products), B Strong arm cuffs; systolic blood pressure minus 20 mmHg, systolic blood pressure plus 110 mmHg, respectively	WR cuff increased BP/RPE more than SEP BFR; SEP BFR generated similar levels of blood lactate responses to the WR cuff condition and rated as more tolerable
Chen et al. (2025a)	2-week RCT (n = 26)	Young adults	Arm curls	WR cuff (GF Health Products), B Strong arm cuffs; systolic blood pressure minus 20 mmHg, systolic blood pressure plus 110 mmHg, respectively	Both arms gained strength; narrow-elastic improved FMD (+2.1%), WR decreased (−1.1%)
Landers et al. (2025)	Acute study (n = 18)	Healthy adults	Arm/leg cycling	B Strong arm and leg cuffs; 160 and 300 mmHg, respectively	BFR group showed trend toward greater tPA increase (fibrinolysis), medium effect size
Liu et al. (2025)	4-week RCT (n = 26)	Athletes with lower back pain	Core exercises	B Strong arm and leg cuffs; 180 for both	Low-load BFR reduced pain and improved core strength similarly to high-load RT
Rolnick et al. (2025a)	Acute crossover (n = 26)	RT adults (n = 25); one non-RT adult	Wall squats	Delfi PTS, B Strong leg cuffs; 60% LOP, 300 mmHg, respectively	Lower body SC-BFR reduced reps and increased discomfort; SEP/MC-BFR matched N-BFR in comfort and performance; no PWV differences observed
Cintineo et al. (2024)	6-week RCT (n = 54)	Army ROTC cadets	Resistance training	B Strong arm and leg cuffs; variable pressures used	MIN + BFR required higher exertion but achieved similar adaptations to traditional training
Rolnick et al. (2024)	Acute crossover (n = 26)	RT adults	Biceps curls	Delfi PTS, B Strong arm cuffs; 60% LOP, 200 mmHg, respectively	Upper body SEP/MC-BFR allowed more reps than SC-BFR; SC blunted arterial stiffness increase
Zhou et al. (2024)	8-week RCT (n = 26)	University athletes	Plyometric/resistance	B Strong leg cuffs; 200 mmHg for the first 4 weeks, 220 mmHg for the second 4 weeks	Power output increased more with BFR complex training vs. control
Dancy et al. (2023)	Acute crossover (n = 42 arms)	Healthy adults	Biceps curls	Delfi PTS, SmartCuffs, B Strong arm cuffs; 50% LOP, 50% LOP, 200 mmHg, respectively	All BFR devices reduced reps ∼50%; Delfi had highest RPE vs. B Strong/B3
Machek et al. (2023)	Acute crossover (n = 20)	Healthy males	Passive occlusion	Hokanson, B3 leg cuffs; 300 mmHg max pressure, 500 mmHg max pressure, respectively	SEP BFR cuffs produced similar blood flow reductions as rigid at equivalent %AOP; first and only study to achieve AOP in SEP B3 cuff
Montoye et al. (2023)	Validation study (n = 23)	Healthy adults	LOP determination	5–13 cm rigid and SEP B Strong leg cuffs; 149–179 mmHg, unable to determine LOP in B Strong cuff	Cuff width affects LOP determination; wider cuffs required lower pressure for occlusion
Wang et al. (2023)	4-week RCT (n = 16)	Young swimmers	Back squats	B Strong leg cuffs; 200 mmHg	Leg strength improved similar to high-load RT; no excessive cardiac strain
Zhang et al. (2023)	Acute crossover (n = 16)	Sprinters	Sprint + WBV	B Strong leg cuffs; 200–350 mmHg	All BFR conditions improved sprint times; sprint + WBV + BFR showed greatest EMG/lactate
Callanan et al. (2022)	Acute crossover (n = 15)	Active individuals	VersaClimber	B Strong arm and leg cuffs applied simultaneously; pressures not reported	Both groups improved VO_2_max similarly; no difference in inflammatory markers
Citherlet et al. (2022)	Acute crossover (n = 11)	Healthy adults	Passive/active	B Strong arm and leg cuffs at 200, 250, 300, 350, and 400 mmHg	B Strong/B3 didn't show pressure-dose differences; couldn't fully occlude at 400 mmHg
Machek et al. (2022)	4-week RCT (n = 18)	RT adults	Leg press	B3 leg cuffs at 80% AOP	Both groups increased leg press strength with BFR; serum ∆IGF-1 was higher in betaine *versus* placebo and serum ∆HCY was greater in high load relative to BFR
Sullivan et al. (2025)	Acute crossover (n = 15)	Female distance runners	Running	B Strong leg cuffs; pressure determined from rigid cuff’s 60% AOP (233 ± 25 mmHg)	LI-BFR running increased lactate/RPE to HI levels despite lower VO_2_; HR intermediate
Wang et al. (2022)	8-week RCT (n = 18)	Volleyball players	Half-squats	B Strong leg cuffs at 180 mmHg	70% 1RM + BFR showed greatest squat/jump gains; 30%1RM + BFR better than 30% 1RM alone
Wooten et al. (2022)	Cohort study (n = 92)	Abdominal cancer patients	Multimodal prehab	B Strong arm and leg cuffs; variable pressures used	BFR prehab: 25% shorter hospital stay, fewer complications vs. standard care
Bordessa et al. (2021)	Crossover RCT (n = 34)	Healthy adults	Knee extensions	Delfi PTS, B Strong leg cuffs; 80% LOP, variable pressures used for B Strong cuff	Pain/RPE highest with rigid BFR; quadriceps EMG similar between devices
Wilburn et al. (2021)	Case report (n = 1)	Apparently healthy male with 13 years RT experience	Leg press	B3 leg cuff at 80% AOP	MRI showed alterations in muscle ultrastructure (wave-like) from BFR
Wooten et al. (2021)	Pre-post (n = 24)	Abdominal cancer patients	Multimodal prehab	B Strong arm and leg cuffs; variable pressures used	BFR prehab increased lean mass and reduced fat mass without weight change; improved 6MWT, chair stand, and TUG; no change in grip strength
Early et al. (2020)	8-week RCT (n = 31)	Healthy adults	Resistance training	B Strong arm and leg cuffs; 250 and 350 mmHg, respectively	SEP BFR produced similar strength gains to traditional; improved FMD
Stray-Gundersen et al. (2020)	Acute crossover (n = 15)	Recreational athletes	Walking	Hokanson, B Strong leg cuffs; 160 mmHg and 300 mmHg, respectively	Rigid cuff significantly increased BP/HR/lactate/RPE; elastic not different from control
Wooten et al. (2020)	Acute crossover (n = 20)	Healthy adults	Yoga	B Strong leg cuffs; 250–300 mmHg	BFR yoga increased lactate without altering RPE; arterial stiffness decreased similarly in both conditions; no FMD change or additive hemodynamic effect of BFR

Abbreviations: AOP, arterial occlusion pressure; BFR, blood-flow restriction; BP, blood pressure; CSA, cross-sectional area; EMG, electromyography; F = female; FMD, flow-mediated dilation; HCY, homocysteine; HI, high-intensity; HR, heart rate; IGF-1, insulin-like growth factor 1; LBP, low back pain; LI, low-intensity; LOP, limb occlusion pressure; MC, multi-chamber; MRI, magnetic resonance imaging; MVC, maximal voluntary contraction; MWT, minute walk test; PE, pulmonary embolism; RCT, randomized controlled trial; RPE, rating of perceived exertion; RT, resistance training; SC, single-chamber; SEP, semi-elastic pneumatic; tPA, tissue plasminogen activator; TUG, timed-up-and-go; VTE, venous thromboembolism; WBV, whole-body vibration.

## 4 Discussion

Recent expert recommendations have emphasized the importance of methodological rigor and standardization in BFR research and practice (Loenneke et al., 2025; Patterson et al., 2019; Rolnick et al., 2023). Patterson et al. established consensus guidelines for the safe and effective prescription of BFR exercise, recommending individualized pressure calibration, appropriate reporting of cuff characteristics, and consideration of participant-specific factors such as limb size and blood pressure (Patterson et al., 2019). Loenneke et al. have also provided updated perspectives on optimizing BFR protocols, including device considerations, application procedures, and outcome monitoring to further enhance safety, efficacy, and best practices across populations and studies (Loenneke et al., 2025). Collectively, these recommendations call for continued refinement of BFR methodologies and transparent reporting in future research. These guidelines underscore the need for methodological rigor in BFR, yet they also highlight a critical gap: current frameworks often do not fully account for SEP BFR devices. The sections that follow attempt to address this gap by outlining key considerations specific to SEP BFR, arguing for its inclusion in future research despite its divergence from AOP-based approaches.

### 4.1 SEP BFR prescription, application, and standardization

SEP BFR cuffs rely on manufacturer-recommended pressures with the ability to adjust pressure based on the ability to complete a set number of repetitions (e.g., 15–30 repetitions), perceptual responses, and rates of fatigue. This can introduce variability—a given pressure may cause more or less perturbation of homeostasis across individuals, depending on factors such as limb size, vascular characteristics, and fitness level. Montoye et al. showed that AOP varied significantly with cuff width and individual characteristics, and the narrowest SEP BFR (B Strong) cuff could not occlude flow in large limbs (Montoye et al., 2023). Importantly, pressure requirements vary among users based on limb size, muscle mass, and training objectives (Patterson et al., 2019). For example, a very muscular individual might need the upper end of the manufacturer’s pressure range (e.g., 350–400 mmHg for legs) to generate an adequate restrictive BFR stimulus, whereas a smaller less muscled individual may require a lower pressure range (150–200 mmHg) to evoke a similar restrictive stimulus. Since SEP BFR users cannot measure AOP directly, one practical approach is to use a familiarization session to identify the pressures required to meaningfully accelerate fatigue, with subsequent adjustments made thereafter. Practitioners and researchers should look for signs of adequate BFR stimulus, such as rapid fatigue onset, venous distension, purple hue in limb, and high RPEs at the end of the latter sets, then increase the pressure accordingly if the starting pressure does not elicit these symptoms.

Another consideration when using SEP BFR cuffs is the initial fitting pressure, or the tightness of the cuff on the limb prior to inflation. Practitioners and researchers seeking to standardize the application can pre-inflate the cuff to ∼25 mmHg before securing it to the limb. Then, while still connected to the pump, one can monitor the increase in pressure as the cuff is tightened, aiming for a fitting pressure between 25 and 50 mmHg. This simple adjustment can improve standardization across users and enhance reproducibility in studies. In addition, researchers and practitioners using SEP BFR devices can monitor metrics like RPE, repetitions to failure, or even NIRS-derived muscle deoxygenation rates to ensure a meaningful BFR training stimulus. One potential approach would be to fit the SEP BFR cuffs at the target pressure, complete 1–2 sets of 30 repetitions, and use real-time NIRS feedback to confirm that muscle oxygen saturation falls into a target range (e.g., 20%–30%) by the end of a given set. NIRS is a promising tool in this instance as it can measure muscle oxygen saturation in real time, which may be useful to quantify the hypoxic stimulus and rates of fatigue across individuals. While some (Willis et al., 2018) have used NIRS to assess muscle and cerebral oxygenation during BFR training, future research should continue to explore differences in rates of fatigue and degree of hypoxia between cuff types and exercises.

### 4.2 Molecular mechanisms and signaling pathways

As fatigue monitoring is crucial for studying BFR and maximizing its effectiveness, it is important to discuss the molecular signaling pathways triggered by the internal stress elicited by BFR exercise. VEGF represents a primary angiogenic signaling factor upregulated by BFR training. Li et al. conducted a meta-analysis revealing that BFR exercise significantly increased mRNA expression (SMD: 0.93, p < 0.05), with resistance exercise showing superior effects over aerobic exercise (Li et al., 2022). Additionally, Ferguson et al. demonstrated that low-load resistance exercise with BFR induced a 5.2-fold increase in VEGF mRNA at 2 h post-exercise, accompanied by enhanced p38MAPK phosphorylation—a key regulator of VEGF transcription (Ferguson et al., 2018). The VEGF response appears linked to local hypoxia created by venous pooling, muscle contraction, and metabolite accumulation (Ferguson et al., 2018; Larkin et al., 2012). The hypoxic stimulus activates hypoxia-inducible factor-1α (HIF-1α), which serves as the primary transcriptional regulator of VEGF expression (Hellwig-BüRgel et al., 1999; Semenza, 2006). In addition, PGC-1α serves as a master regulator of mitochondrial biogenesis and oxidative metabolism (Puigserver and Spiegelman, 2003; Scarpulla, 2011). Meta-analytic evidence indicates that four of five studies showed higher PGC-1α mRNA expression during BFR exercise (SMD: 0.74, p < 0.05) (Li et al., 2022). Peak PGC-1α responses occur 2–4 h post-exercise and mediate downstream expression of nuclear respiratory factors, mitochondrial transcription factor A, and VEGF itself (Ferguson et al., 2018; Larkin et al., 2012). The PGC-1α response to BFR appears driven by multiple stimuli including metabolic stress (elevated AMP:ATP ratio activating AMPK) (Cantó et al., 2009; Jäger et al., 2007), reactive oxygen species (ROS) production, and changes in calcium signaling from enhanced motor unit recruitment (Nielsen et al., 2012; Powers and Jackson, 2008). This multifactorial activation explains how low-load BFR can stimulate mitochondrial adaptations typically requiring high-intensity exercise.

A recent meta-analysis in the American Journal of Physiology (2025) examined HIF-1α responses to exercise, finding that HIF-1α protein levels significantly increase following dynamic exercise (Aragón-Vela and Casuso, 2025). However, the relationship between HIF-1α stabilization and downstream VEGF expression remains complex and appears modulated by exercise intensity, duration, and the degree of hypoxia achieved. Nonetheless, SEP BFR can serve as a useful tool to enhance the rate and degree of tissue hypoxia to activate HIF-1α and promote angiogenesis during low-intensity exercise. Current evidence presents competing theories regarding primary metabolic triggers for BFR adaptations (Hughes et al., 2017; Scott et al., 2015). The lactate accumulation hypothesis suggests that reduced venous outflow in combination with exercise accelerates metabolite accumulation, with lactate serving as both a metabolic substrate and signaling molecule to activate mammalian target of rapamycin (mTOR) pathways (Suga et al., 2009; Takano et al., 2005). Supporting this, acute BFR studies consistently show a 3- to 5-fold increase in blood lactate comparable to high-intensity exercise (Shimizu et al., 2016; Yasuda et al., 2006). Alternatively, the ROS hypothesis proposes that repeated hypoxia-reperfusion cycles activate redox-sensitive transcription factors including NF-κB and AP-1 (Powers and Jackson, 2008). Triggering these pathways serve to upregulate antioxidant defenses, mitochondrial biogenesis, and other angiogenic factors.

### 4.3 Safety and risk management

When appropriate pre-screening and protocols are followed, the reported complications from BFR training are generally minor (numbness, petechiae, headache, syncope, *etc.*) and severe complications (excessive pain, central retinal vein occlusion, and rhabdomyolysis) are rare (Anderson et al., 2022; Nascimento et al., 2022; Patterson and Brandner, 2018). In addition, established BFR guidelines recommend using sub-occlusive pressures to reduce the risk of adverse cardiovascular events and excessive peripheral nerve compression (Nascimento et al., 2022; Patterson et al., 2019; Stavres et al., 2018). In fact, a major rationale for AOP calibration is to avoid complete occlusion during training to mitigate complications arising from ischemia during exercise. However, the ability of SEP BFR cuffs to expand with the limb during muscle contraction and not occlude arterial flow may provide enhanced safety and comfort. Nevertheless, standard BFR safety precautions apply regardless of cuff type. Absolute contraindications include uncontrolled hypertension, peripheral arterial disease, active deep vein thrombosis, pregnancy, and sickle cell disease (Nascimento et al., 2022) while relative contraindications requiring medical clearance include controlled hypertension, diabetes, and obesity. Supervision or guidance by trained personnel is advised for high-risk and clinical populations. Proper application (placement at proximal portion of the limb, appropriate fitting pressures, gradual pressure progression strategies, *etc.*) as well as adherence to suggested exercise durations (e.g., <20 min at a time) further promote safety. Overall, a common-sense approach should address the vast majority of safety concerns related to SEP BFR and BFR training as a whole.

### 4.4 Implementation and accessibility

One reason SEP BFR has proliferated in the real-world is its accessibility—SEP BFR devices like B Strong/B3 are commercially available and relatively easy to use. As more practitioners implement BFR, it will be important to disseminate best practices specific to SEP BFR cuffs. For example, instructional guidelines might emphasize that users focus on reaching high exertion (e.g., performing three out of four sets to volitional failure or near-failure). Importantly, a gradual approach to increasing the pressure is advised when using any BFR system. Also, because SEP BFR cuffs allow simultaneous arm and leg training, practitioners are applying full body BFR during exercise (Callanan et al., 2022; Landers et al., 2025). More studies investigating the effects of whole-body SEP BFR would advance the broader BFR literature and represent a unique aspect of SEP BFR application. In addition, passive forms of BFR, including the use of neuromuscular electrical stimulation (NMES) combined with BFR represents a rapidly growing area of research interest with significant clinical and rehabilitative potential. While studies have been performed using AOP-based devices with NMES (Head et al., 2021; Rice et al., 2023; Santiago-Pescador et al., 2023), no studies have investigated the combination of SEP BFR with NMES. As mentioned previously, future SEP BFR cuffs might incorporate built-in NIRS sensors to monitor muscle oxygenation levels to ensure an effective stimulus in real-time. Moreover, integration with virtual applications could also allow users to log their session, pressures used, RPE, proximity to failure, and number of repetitions achieved.

### 4.5 Comparative research

Relatively few studies have directly compared the efficacy of SEP and AOP-calibrated BFR cuffs. A notable exception includes Chen, McLaurin et al. who found divergent vascular effects between NE/SEP and non-AOP-calibrated WR cuffs likely stemming from increased retrograde shear stress in the WR condition (Chen L.-S. et al., 2025). Dancy et al. found no differences in exercise performance, RPE, or safety across three commonly-used BFR cuffs (B Strong, SmartCuffs, and Delfi PTS) during resistance exercise (Dancy et al., 2023). In addition, Rolnick et al. have made several direct comparisons between SC AOP-calibrated cuffs to MC/SEP BFR cuffs (Rolnick, 2025; Rolnick et al., 2024) and found a lack of increase in central arterial stiffness in the rigid AOP-based SC cuffs compared to MC/SEP BFR cuffs and unrestricted resistance exercise (Rolnick et al., 2024). Considering the variability in findings, more direct comparison trials are needed to determine the relative safety and efficacy in the short- and long-term. It will also be informative to compare different SEP BFR devices (e.g., KAATSU vs. B Strong/B3) as well as different iterations of the same device.

Given that SEP BFR is relatively new in research, we anticipate a wave of upcoming studies examining muscular signaling pathways activated by SEP *versus* rigid AOP-calibrated BFR cuffs as well as specific adaptations in a variety of populations. Mechanistic research can further elucidate how BFR training induces adaptations and whether there are differences between devices and/or traditional training conditions. Preliminary evidence suggests that SEP and rigid non-AOP-calibrated BFR cuffs induce different vascular shear stress profiles (Chen L. et al., 2025). While these findings may be due to the lack of autoregulation and/or AOP-based calibration, these vascular responses may also suggest that cuff types differentially modulate the expression of angiogenetic pathways such as VEGF or PGC-1α. Future research priorities include: 1) standardized protocols comparing muscle protein synthetic rates, gene expression profiles, and chronic adaptations between intensity-matched SEP and AOP-based BFR cuff groups/conditions; 2) investigation of optimal pressure ranges for specific populations, particularly clinical groups where safety considerations are paramount; and 3) development of objective fatigue indicators (e.g., NIRS) that can standardize the dose of BFR independent of cuff type or BFR methodology.

Finally, bridging the gap between the AOP-centric research community and the practical SEP BFR user community is paramount. Rather than viewing them in opposition, future work should aim to identify how both approaches can inform each other. Ultimately, both types of cuffs are tools to apply a BFR stimulus. An open-minded, evidence-driven approach is needed to progress this rapidly evolving field.

## 5 Limitations

While this narrative review offers important insights into the use and application of SEP BFR devices, several limitations should be acknowledged to contextualize its findings. First, pressure prescription variability remains a substantial limitation across SEP BFR research. Studies report pressures ranging from 150 to 400 mmHg and are sometimes applied uniformly without individualization for limb circumference, body composition, or fitness level. Future research should incorporate standardized and transparent reporting of cuff specifications to enable meaningful analyses and practical translation. Another limitation is the paucity of direct head-to-head comparisons between AOP-based and SEP BFR cuffs. Currently, only acute or short-term studies comparing devices have been performed, necessitating longitudinal comparison studies investigating the potential differences in adaptations between cuff types. Along these lines, due to the relative dearth of studies utilizing SEP BFR cuffs, there is a lack of protocol consistency between trials examined in this review (e.g., exercise type, intensity, populations, *etc.*), which limits the strengths of our conclusions. More rigorous longitudinal comparisons utilizing similar exercise protocols, cuff pressures, larger sample sizes, and longer-term follow-ups are paramount.

Future trials should also seek to isolate device-specific effects on both acute physiological responses and chronic training adaptations. Sex and race representation also remain critically inadequate. Most studies recruit predominantly young male participants, with women comprising only 20%–40% of recent cohorts. The limited evidence suggests potential sex differences in discomfort perception and hemodynamic responses (Spitz et al., 2021), yet small sample sizes preclude definitive conclusions. Future research should seek to implement inclusive recruitment strategies following SAGER guidelines, with *a priori* power calculations for sex-stratified analyses and transparent reporting of participant demographics to enhance generalizability across diverse populations. In addition, individual response heterogeneity, while acknowledged, is rarely analyzed systematically. Variability in acute hemodynamic responses and chronic adaptations likely reflects complex interactions among limb size/composition, fitness level, and pain thresholds. Preliminary mechanistic insights from resistance training studies suggest metabolic stress and proximity to failure may drive adaptations, but these mechanisms remain poorly characterized.

## 6 Conclusion

This narrative review sought to provide a focused, evidence-based perspective on SEP BFR training. While many reviews address BFR more broadly, few, if any, have offered an in-depth analysis specific to SEP BFR devices. Our aim was threefold: 1) highlight the acute and chronic physiological responses associated with SEP BFR; 2) identify key methodological considerations unique to SEP BFR devices; and 3) encourage further mechanistic and comparative research. Limited evidence suggests that while SEP BFR devices lack the ability to provide individualized AOP calibration, they can nevertheless elicit significant improvements in muscle strength, hypertrophy, endurance, and vascular function across diverse populations. Moreover, these adaptations are achieved while maintaining a high degree of safety and accessibility. SEP BFR cuffs promote the key stimuli that drive adaptation: fatigue, metabolite accumulation, and enhanced motor unit recruitment. These features, coupled with their accessibility and ease of use, make SEP BFR a viable and scalable tool in both clinical and performance settings.

Importantly, this review was conceived in the context of a growing emphasis in the literature on standardizing BFR training through AOP-based calibration. While precision is valuable—especially in research and clinical environments—this emphasis should not preclude other effective forms of BFR from scientific inquiry. SEP BFR devices, modeled after the original KAATSU method, offer a practical and scalable BFR system that aligns closely with the foundational principles of BFR training. Rather than dismissing SEP BFR devices due to their inability to fully occlude, it is constructive to recognize that true physiological standardization is elusive even when using AOP-based methods. Thus, over-reliance on pressure as the primary variable may detract from other relevant drivers of adaptation. Moving forward, researchers are encouraged to study SEP BFR with the same rigor given to AOP-based BFR devices, using carefully controlled designs to explore which cuffs and protocols might be best indicated for a given application or population. At the same time, ongoing comparisons and integration with AOP-based knowledge will strengthen the overall understanding of BFR.

In conclusion, SEP BFR training appears to provide a safe, effective, and scalable approach to BFR training. The current literature supports its efficacy in improving a range of health and performance outcomes. With further research, especially direct cuff comparisons, we can refine guidelines for BFR training to maximize its translation to real-world application. The future of BFR training should embrace both the high-precision approach of AOP-calibrated systems and the real-world practicality of SEP BFR devices. Ultimately, pressure is just one variable; it is the intelligent application of BFR to create a fatiguing stimulus that confer results. Continued scientific inquiry and open collaboration between proponents of different methods will advance BFR training to benefit a wide spectrum of populations and applications.
